# Supplementary material for: Effects of older age on contraction-induced intramyocellular acidosis and inorganic phosphate accumulation in vivo: A systematic review and meta-analysis
Source: PLoS One. 2024 Sep 25;19(9):e0308336. doi: 10.1371/journal.pone.0308336 (PMC11424002; doi:10.1371/journal.pone.0308336)
Supplement: S2 Appendix — (PDF) [file pone.0308336.s002.pdf]

S2 Appendix.

PubMed Search:
Search terms 1: (contract* OR exercise OR “muscle activation” OR eccentric), 2: (acidosis OR pH OR proton OR hydrogen ion), 3: (aging OR old OR older OR elder* OR senior OR age OR aged OR geriatric OR gerontology OR geroscience), 4: (MRS OR nuclear magnetic resonance OR NMR OR phosphorus OR MR spectroscopy), 5: (muscle)
6: 1 and 2 and 3 and 4 and 5

Web of Science and SPORTDiscus Search
Search terms 1: (contract* OR exercise* OR “muscle activation” OR eccentric*), 2: (acidosis OR pH OR proton* OR hydrogen ion*), 3: (aging OR old* OR elder* OR senior* OR age* OR geriatric* OR gerontology OR geroscience), 4: (magnetic resonance spectroscopy OR MRS OR nuclear magnetic resonance OR NMR OR phosphorus OR MR spectroscopy), 5: (muscle*)
6: 1 and 2 and 3 and 4 and 5
