# Supplementary material for: Effects of older age on contraction-induced intramyocellular acidosis and inorganic phosphate accumulation in vivo: A systematic review and meta-analysis
Source: PLoS One. 2024 Sep 25;19(9):e0308336. doi: 10.1371/journal.pone.0308336 (PMC11424002; doi:10.1371/journal.pone.0308336)
Supplement: S3 Appendix — (PDF) [file pone.0308336.s003.pdf]

S3 Appendix.

Newcastle-Ottawa Quality Assessment Scale Modified for Cross-Sectional Studies (adapted from Krüger et al. 2018)

Selection (4-star maximum):

1) Representativeness of the sample:

a) Representative of the average in the target populations. *

b) Selected group of participants from local groups, university populations, or no description of the sampling strategy.

2) Sample size.

a) Justified and satisfactory. *

b) Not justified.

3) Ascertainment of participants' health status.

a) Validated measurement tool of health status, such as medical report, medical history form, PAR-Q questionnaire. **

b) reported health status. *

c) No description of participants' health status.

Comparability (2-star maximum):

1) Comparability of age groups based on controlling for confounding factors.

a) The study controls for physical activity with a validated measurement tool, such as an accelerometer, a self-report tool, or described by the authors. *

b) The study controls for any additional factors (e.g. caffeine, alcohol, and/or exercise prior to testing). *

c) a & b.**

d) No description.

Outcome (3-star maximum):

1) Assessment of the main outcome:

a) Validated equation for pH is referenced/used. **

b) No description.

2) Statistical test:

a) The statistical test used to analyze the data is clearly described and appropriate, and the probability level and/or effect size is reported. *

b) The statistical test is not appropriate, not described, or incomplete.
