# Supplementary figures and images for: Effects of older age on contraction-induced intramyocellular acidosis and inorganic phosphate accumulation in vivo: A systematic review and meta-analysis
Source: PLoS One. 2024 Sep 25;19(9):e0308336. doi: 10.1371/journal.pone.0308336 (PMC11424002; doi:10.1371/journal.pone.0308336)

S1 Fig.

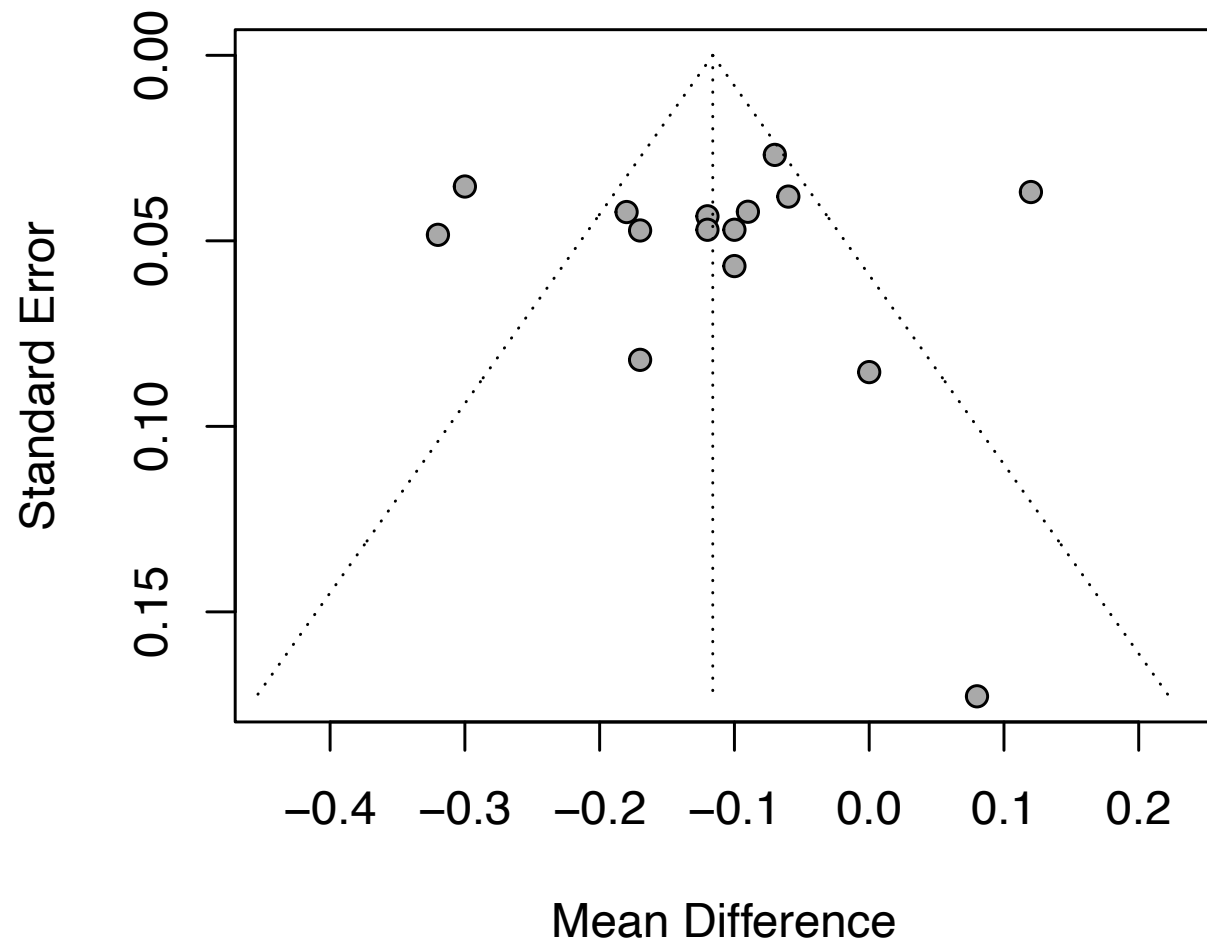

Supplement: S1 Fig — The mean difference is plotted on the x-axis and the standard error on the y-axis. Individual studies are represented by gray circles (k = 12, 15 effects). The vertical dashed line represents the overall effect for age-related differences in pH in response to standardized contractions and the diagonal dashed lines represent the 95% confidence interval. Symmetrical distribution of studies and a non-significant Egger’s test (p = 0.446) suggest there is no publication bias. (PDF) [file pone.0308336.s004.pdf]

S2 Fig.

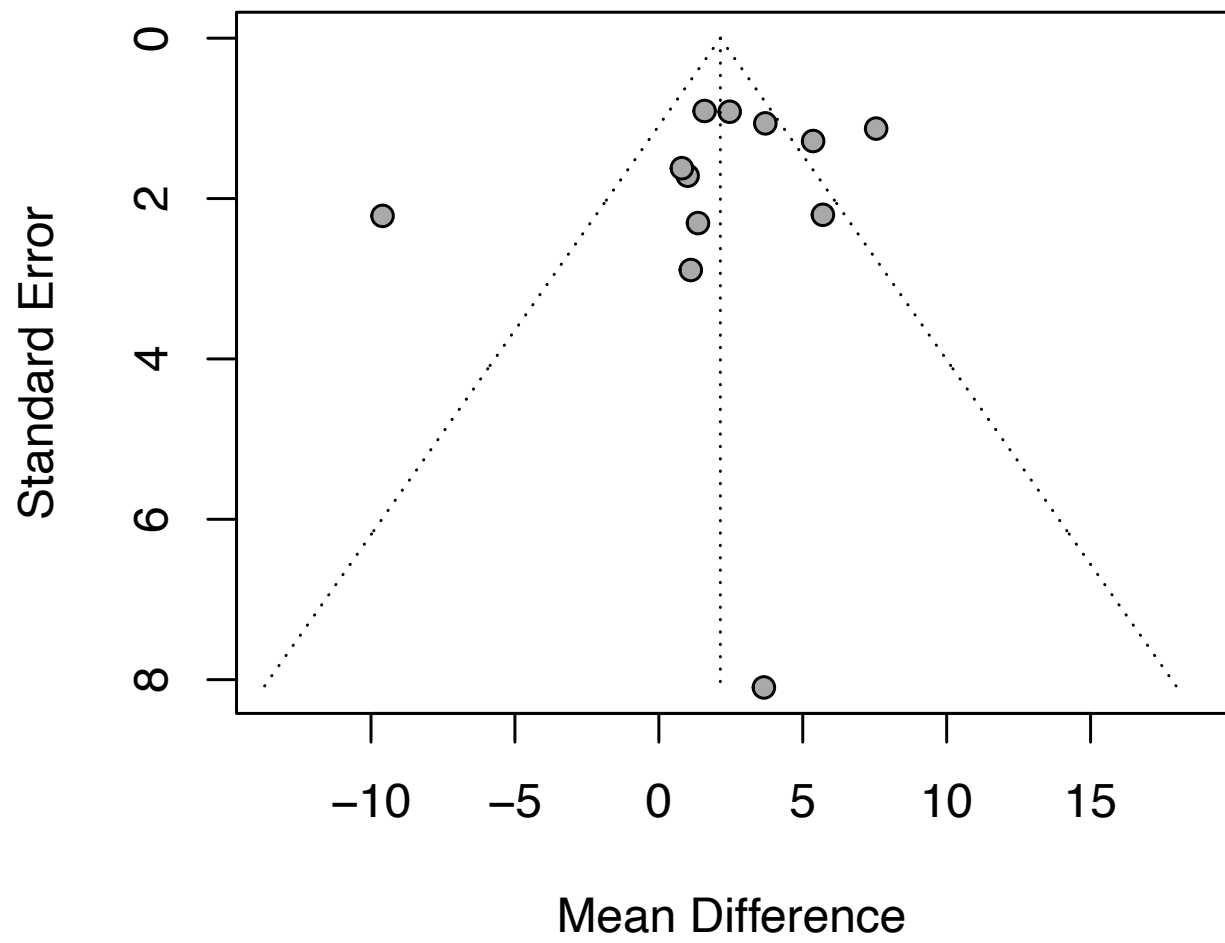

Supplement: S2 Fig — The mean difference is plotted on the x-axis and the standard error on the y-axis. Individual studies are represented by gray circles (k = 9, 12 effects). The vertical dashed line represents the overall effect for age-related differences in [Pi] in response to standardized contractions and the diagonal dashed lines represent the 95% confidence interval. Symmetrical distribution of studies and a non-significant Egger’s test (p = 0.122) suggest there is no publication bias. (PDF) [file pone.0308336.s005.pdf]

S3 Fig.

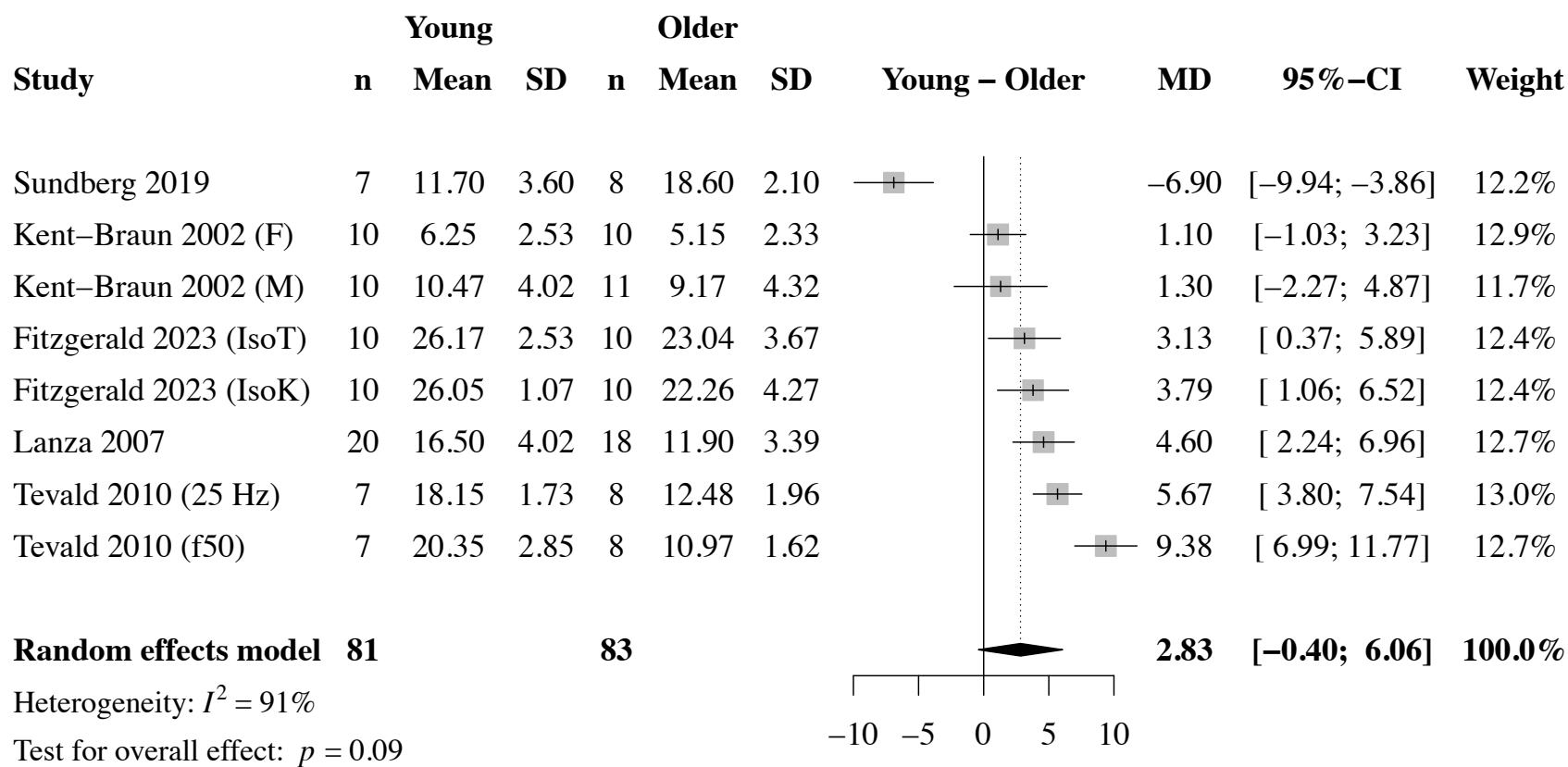

Supplement: S3 Fig — Forest plot for the overall effect of age on intramyocellular diprotonated inorganic phosphate (H2PO4-) in response to standardized muscle contractions (k = 5, 8 effects). A positive value represents greater [H2PO4-] in young compared with older muscle. Overall, there was no difference in end-exercise [H2PO4-] between young and older muscle. However, a sensitivity analysis using the leave-one-out method revealed that when Sundberg et al. [14] was removed, younger muscle had greater intramyocellular [H2PO4-] compared with older muscle (MD = 4.23 mM; 95% CI = 2.11, 6.35; p < 0.01; I2 = 81%; k = 4; 7 effects). (PDF) [file pone.0308336.s006.pdf]
