# Supplementary material for: Effects of older age on contraction-induced intramyocellular acidosis and inorganic phosphate accumulation in vivo: A systematic review and meta-analysis
Source: PLoS One. 2024 Sep 25;19(9):e0308336. doi: 10.1371/journal.pone.0308336 (PMC11424002; doi:10.1371/journal.pone.0308336)
Supplement: S1 Table — Quality assessment scores for each study from the Newcastle-Ottawa Quality Assessment Scale Modified for Cross-Sectional Studies [7, 37]. This scale assesses the quality of studies based on a total of nine stars across three sections: Selection (four stars), Comparability (two stars), Outcome (three stars). The Risk of Bias analysis rated each study in each section as “good”, “fair”, or “poor” [38], which are represented as green, yellow, and red, respectively. (PDF) [file pone.0308336.s007.pdf]

S1 Table.

	Selection			Comparability	Outcome		Total
**Study**	1	2	3	1	1	2	
Taylor 1997			*		**	*	4
Smith 1998			**	*	**	*	6
Chilibeck 1998					**		2
Kutsuzawa 2001			*		**		3
Kent-Braun 2002	*		*	*	**	*	6
Lanza 2005			*	**	**	*	6
Lanza 2007			*	**	**	*	6
Tevald 2010			*	**	**	*	6
Layec 2014			*	**	**	*	6
Layec 2015		*	*	**	**	*	7
Sundberg 2019			**		**	*	5
Fitzgerald 2023			**	*	**	*	6

Risk of Bias analysis was completed based on the scoring algorithm by McPheeters et al. 2012 (https://www.ncbi.nlm.nih.gov/books/NBK107322/). Green = “good”, yellow = “fair”, red = “poor”.
