# Supplementary material for: Effects of older age on contraction-induced intramyocellular acidosis and inorganic phosphate accumulation in vivo: A systematic review and meta-analysis
Source: PLoS One. 2024 Sep 25;19(9):e0308336. doi: 10.1371/journal.pone.0308336 (PMC11424002; doi:10.1371/journal.pone.0308336)
Supplement: Attachment — Submitted filename: PLOS ONE March 2024.pdf [file pone.0308336.s008.pdf]

EFFECTS OF OLD AGE ON CONTRACTION-INDUCED INTRAMYOCELLULAR ACIDOSIS AND INORGANIC PHOSPHATE ACCUMULATION IN VIVO: A SYSTEMATIC REVIEW AND META-ANALYSIS

Arieta LR, Smith ZH, Paluch AE, Kent JA (PONE-D-24-07597)

GENERAL COMMENTS

This systematic review and meta-analysis describes the changes of intracellular pH and inorganic phosphate that occur with fatiguing exercise in aging human muscle versus younger adult muscle. There is also a particular interest in comparing these metabolic changes in static versus dynamic contractions to investigate whether they relate to age-related differences in fatigue resistance with these contraction modes. These are pertinent issues presented with sound rationale. This is a well performed and straightforward piece of work that reads well. My suggestions below should strengthen interest in this work.

MAJOR COMMENTS

The authors present mean difference for comparing pHi and Pi between groups. It would be helpful for many readers to also see the absolute values (mean with SD) either in the text or figures. Furthermore, this is important because the same mean pH difference, given as absolute [H^+], can be quantitatively different at different pH levels.

It would also be extremely valuable for the authors to calculate and evaluate differences in diprotonated Pi between groups given the widespread interest in this species. This would bolster interest in this work.

Were there any differences in resting pHi and Pi between groups? If so this could indicate different H^+ regulating processes at rest.

Page 15: The Sundberg et al. 2019 study is well-performed (mainly very old female) as is the

study by Fitzgerald et al. 2023. The discussion and treatment of these studies, given different responses, needs greater consideration. The difference is postulated to be due to posture differences. Please explain the putative mechanism underpinning this postural effect as it is unclear?

MINOR COMMENTS

The authors should briefly acknowledge other metabolic changes (and other fatigue mechanisms) that are likely to occur during the exercise protocols of the present review.

The authors interchange use of intramyocellular and intracellular – it would be better to consistently use one expression.

EDITOR

This is a novel, valuable and well-performed study satisfying the journal criteria. There is some useful information on measures of pHi and Pi in exercising muscles of younger and older adults, especially in relation to contraction mode. Incorporating [$H_2PO_4^-$] measures in young and old as well would considerably bolster interest in this work. The news is certainly worthy of publication once the comments have been addressed.
