# Supplementary material for: Effects of older age on contraction-induced intramyocellular acidosis and inorganic phosphate accumulation in vivo: A systematic review and meta-analysis
Source: PLoS One. 2024 Sep 25;19(9):e0308336. doi: 10.1371/journal.pone.0308336 (PMC11424002; doi:10.1371/journal.pone.0308336)
Supplement: Attachment — Submitted filename: Review_PONE-D-24-07597.pdf [file pone.0308336.s009.pdf]

The present study aimed to systematically review and meta-analyze studies regarding the effects of age (young vs. old) on intramyocellular acidosis and inorganic phosphate accumulation in vivo. The authors focused on cross-sectional responses to standardized skeletal muscle protocols such as dynamic and isometric contraction trials comparing these responses between young (18-45 yr.) and old (55+ yr.) individuals. Overall, the manuscript is well written, the methods and analysis for the systematic review and meta-analysis are adequate to answer the research question and, the conclusions are in line with the obtained results. I have, nevertheless, some minor comments that I would like to address before fully supporting the publication of the manuscript.

1. Abstract:
    a. In the abstract, the authors mention "**Young muscle acidified more than older muscle**". Although I understand the whole idea, the reality is that is not the muscle that generates the acidification, but the metabolic acidosis is the consequence of the muscle contraction.
    b. I am unsure about the last sentence as is not clear. Maybe the reader would benefit from a better characterization of what you define as "an advantage during dynamic contractions"?
2. Introduction:
    a. "**The way in which fatigue is measured in response to dynamic contractions, either as muscle torque or power, likely contributes to the equivocal results in the literature to date**". Very good point!
    b. Regarding the line: "**fatigue is measured as the decline in muscle power**", I think fatigue can't be measured. Instead, the decline of muscle power is a proxy measure for a certain type of fatigue.
    c. "**This greater mitochondrial oxidative capacity of older muscle could allow a relative sparing of the need for non-oxidative ATP production during standardized contraction protocols, and thus reduce acidosis and the accumulation of Pi**". I agree with the premise, however, this would probably depend on the type and intensity of contraction (i.e., maximal vs. submaximal).
3. Methods
    a. "**Contraction protocols designed to limit acidosis (e.g., short, "oxidative capacity" protocols)". Please could you elaborate on what you consider oxidative capacity protocols? For instance, what do you consider "short"**? In time, would the metabolic pathway dominance depend also on the intensity of muscle contraction?

b. “studies in which mean intramyocellular pH of at least one group was not ≤ 6.90” I am not fully sure about this criterion. Although I understand the rationale behind it and could agree to some degree, I also feel it could be a double-edged sword. In this scenario, the authors state: “Including studies that did not observe muscle pH ≤ 6.90 could artificially mask age-related differences due to an insufficient perturbation from resting” and thus I wonder if the opposite could also be true…

c. Regarding the quality assessment, in addition to the instrument reported, the authors must present the Risk of Bias analysis per included study.

d. After screening the study characteristics in Table 1 one aspect that caught my attention was the diversity of skeletal muscle groups used in the standardized contraction protocols. It is known that there can be variations in inorganic phosphate levels among different skeletal muscle groups and its levels can vary depending on factors such as muscle fiber type composition. For instance, fast-twitch muscle fibers (Type II fibers) typically have higher levels of Pi compared to slow-twitch fibers (Type I fibers). Consequently, muscles with a higher proportion of fast-twitch fibers may exhibit higher Pi concentrations. Additionally, the distribution of Pi can vary within individual muscles based on factors like motor unit recruitment and muscle architecture. Therefore, based on the previous premises I wonder if there could be differences in the outcome of the meta-analysis when using different skeletal muscle groups. I’ve noticed that you mention some of this in the discussion. However, it would be beneficial for the reader if you elaborate on this issues and how may impact on the results you have obtained with the meta-analysis.

4. Discussion

a. In the subsection Age-related differences in pH and [Pi] you mention that “**Notably, removing the study by Sundberg et al. [14] resulted in significant overall effects for both pH and [Pi]**” However in the results section when referencing the same study you state that “**Removing the same study from the overall effect for pH did not change those results**”. Please clarify.

b. “**This result is in agreement with the overall greater oxidative capacity in older muscle and refutes the concept of a de facto decrease in mitochondrial energy production as a consequence of older age**”. Considering the findings of the systematic review and meta-analysis, I believe that employing the term "refutes" may come across as overly assertive. Perhaps opting for a milder verb would better convey the intended meaning.

c. “**Older muscle may have lower maximal motor unit discharge rates**” I agree, but also older individuals often show reduced fast motor unit and consequently fewer type 2 muscle fibers. The authors discuss this issue in the subsection about “Isometric Contractions” yet, in my opinion, is an issue that deserves being discussed here.

d. “**The relatively high (I2 = 82-86%, all studies) heterogeneity in the overall effects reported here is likely due to the inclusion of studies that used different contraction protocols and modes, muscle groups, and participant physical activity status**”. This is a pivotal point that warrants a more thorough discussion, given that high values of heterogeny could greatly affect the results of the meta-analysis.

e. In the paragraph about the influence of contraction mode, could you elaborate on the meaning of “energetic advantage”?
