# Supplementary material for: Effects of older age on contraction-induced intramyocellular acidosis and inorganic phosphate accumulation in vivo: A systematic review and meta-analysis
Source: PLoS One. 2024 Sep 25;19(9):e0308336. doi: 10.1371/journal.pone.0308336 (PMC11424002; doi:10.1371/journal.pone.0308336)
Supplement: Attachment — Submitted filename: Arieta_AgingMeta_PLOSONE_ResponseToReviewers_R2.docx [file pone.0308336.s011.docx]

PONE-D-24-07597

Effects of old age on contraction-induced intramyocellular acidosis and inorganic phosphate accumulation in vivo: A systematic review and meta-analysis

PLOS ONE

Reviewer #1: GENERAL COMMENTS

As mentioned previously this systematic review and meta-analysis describes the changes of intracellular pH and inorganic phosphate that occur with fatiguing exercise in aging human muscle versus younger adult muscle, which are pertinent issues presented with sound rationale. The further consideration of H2PO4- was a helpful addition – thanks for including this. Overall, this is a well-performed piece of work and the tweaking of the text has been beneficial

COMMENTS

As mentioned previously it would be “helpful” for many readers to also see the absolute values for pHi and Pi between groups (mean with SD) either in the text or figures. This has been argued against. I agree that readers can see the data from the individual studies, and also calculate the mean data across studies from this. The authors need to decide whether they want to be “helpful” or not? Comments “from a statistical standpoint…” do not hold if the authors also show the mean difference for these values which then has statistical issues.

*We have added the mean and SD for the absolute values for pH, Pi, and H2PO4- to the Results section. These have been calculated according to the equation in Table 6.5.a in the Cochrane Handbook (*[*https://training.cochrane.org/handbook/current/chapter-06#section-6-5*](https://training.cochrane.org/handbook/current/chapter-06#section-6-5)*). This information has been added to the methods section.*

The effects of pH and inorganic phosphate on force production appear to be synergistic rather than additive. Like you mention much of the Pi exists as H2PO4- under acidic milieu. Hence the reason to evaluate H2PO4- in young and older muscle, regardless of whether or not it is simply total Pi or H2PO4- that contributes to fatigue mechanisms

Introduction – the authors have now mentioned other potential mechanisms of fatigue (page 5) although it would also be helpful to mention electrolyte shifts and reactive oxygen species (perhaps refer to a review). The comment that these other aspects will contribute to muscle fatigue by a smaller extent than pH and Pi is questionable – there is no doubt that pH and Pi are important bioenergetic contributors to fatigue but quantitative comments have not been justified.

*We have added mention of electrolytes and reactive oxygen species to the other potential mechanisms, which are covered in reference 16. We have also removed the comment “although likely to a smaller extent than pH and Pi” to avoid any quantitative comments.*

It may be wiser to use “older age” rather than “old age” in the title, even given that the authors found no statistical difference for those <65 yr versus the more elderly. For authors consideration only.

*We have made this change, as requested.*

Reviewer #2: I would like to express my gratitude and acknowledge the authors' efforts in addressing all of my comments. All the issues I raised have been thoroughly resolved, and as a result, I recommend the manuscript for publication.

*Thank you.*
